# Newborn Behavioral Observation, maternal stress, depressive symptoms and the mother-infant relationship: results from the Northern Babies Longitudinal Study (NorBaby)

**DOI:** 10.1186/s12888-020-02669-y

**Published:** 2020-06-15

**Authors:** Ragnhild Sørensen Høifødt, Dag Nordahl, Inger Pauline Landsem, Gábor Csifcsák, Agnes Bohne, Gerit Pfuhl, Kamilla Rognmo, Hanne C. Braarud, Arnold Goksøyr, Vibeke Moe, Kari Slinning, Catharina Elisabeth Arfwedson Wang

**Affiliations:** 1 Department of Psychology, Faculty of Health Sciences, UiT The Arctic University of Norway, Tromsø, Norway; 2 Division of Child and Adolescent Health, University Hospital of Northern Norway, Tromsø, Norway; 3 Department of Health and Care Sciences, Faculty of Health Sciences, UiT The Arctic University of Norway, Tromsø, Norway; 4 Department of Social Science, Faculty of Health and Social Science, Western Norway University of Applied Sciences, Bergen, Norway; 5 Regional Center for Child and Youth Mental Health and Child Welfare, NORCE Norwegian Research Centre AS, Bergen, Norway; 6 Department of Psychology, Faculty of Social Sciences, University of Oslo, Oslo, Norway; 7 Regional Centre for Child and Adolescent Mental Health East and South, Oslo, Norway

**Keywords:** Newborn behavioral observation, Intervention, Parenting stress, Postpartum depression, Mother-infant relationship

## Abstract

**Background:**

Families can experience the postpartum period as overwhelming and many report a special need for support. The Newborn Behavioral Observation (NBO) aims to promote a positive parent-infant relationship by sensitising parents to the infant’s signals. This article evaluates the NBO as a universal preventive intervention within the regular well-baby clinic service on measures of maternal depressive symptoms, parental stress, the mother-infant relationship and satisfaction/benefit of the postpartum follow-up.

**Methods:**

This investigation is part of a larger longitudinal study comprising 220 women and 130 of their partners recruited between 2015 and 2017. The study had a non-randomised cluster-controlled design with 6 measurement points. This article is based on a sample of 196 women using data from T1 (gestational weeks 13–39), T4 (5–15 weeks postpartum) and T5 (3–9 months postpartum). Participants were allocated to a group receiving the NBO (*n* = 82) and a care as usual comparison group (*n* = 114). We measured maternal depressive symptoms and parental stress using the Edinburgh Postnatal Depression Scale (EPDS) and the Parenting Stress Index (PSI). The mother-infant relationship was assessed with the Parental Reflective Functioning Questionnaire (PRFQ), the Maternal Postnatal Attachment Scale (MPAS) and the Maternal Confidence Questionnaire (MCQ). Participants also answered questions about satisfaction/benefit of the postpartum follow-up.

**Results:**

A Mann-Whitney *U* test indicated that participants in the NBO-group learned significantly more than the comparison group from the follow-up about the baby’s signals in relation to sleep/sleep patterns, social interaction and crying/fuzziness. Multivariate analyses of covariance (MANCOVA) and repeated measures ANCOVA found no significant differences between the groups for the mother-infant relationship domain and few differences in depressive symptoms and parental stress. The repeated measures ANCOVA found that participants in the NBO-group scored slightly higher on parental stress, although the difference was small.

**Conclusions:**

The results indicate that the NBO-group learned more than the comparison group about reading their child’s signals in important everyday situations. However, the benefits of the NBO were limited for depressive symptoms, parental stress and self-reported mother-infant relationship. The study sample was generally well-functioning, and the results indicate that the benefits of the NBO may be limited within a well-functioning sample.

**Trial registration:**

ClinicalTrials, NCT02538497, Registered 2 September 2015.

## Background

Becoming a parent is a major life transition, and the postpartum period is characterized by large biological and psychosocial changes [1]. Within these first weeks and months, parents start to know their infant, manage child-care tasks and the majority develop confidence and satisfaction in their new roles [2, 3]. However, families can experience the transition as overwhelming and many report a special need for support and care from their social network and professionals [4]. This increased vulnerability of the postpartum period points to the necessity of health care workers to empower families to reduce stress and strain, and to increase their ability to cope with their new circumstances. In addition, since these first months is a period of rapid development for the infant, the parents and the parent-infant relationship, this period serves as a “window of opportunity” during which intervention may contribute significantly in enhancing a positive transition [5].

### Development of the parent-infant relationship

The newborn period entails an important transition for the parent-infant relationship. Newborns are predisposed to interact socially using their gaze, gestures, vocalisations and emotional expressions [6, 7]. Positive parent-infant interactions depend on the caregiver’s ability to respond sensitively to these individually expressed signals [8]. The development of a healthy parent-child relationship and positive parenting is influenced by several factors, including parental well-being and their sense of competence and self-efficacy in caring for the infant [9, 10]. A central concept is parental reflective functioning (mentalization), i.e. the parents’ capacity to understand and reflect upon one’s own and the child’s behaviours as expressions of underlying mental states [11]. Higher maternal reflective functioning is related to more positive maternal caregiving behaviours, especially affective communication [12], and is intrinsically linked to sensitive caregiving [13]. Another important aspect is the emotional bond experienced by the parent towards the child, which can be seen as an affective and cognitive dimension of the parent-child relationship [14]. This bond develops during pregnancy, is fairly stable from pregnancy until toddlerhood [15], and is predictive of maternal sensitivity [16].

Through attuned, sensitive and responsive interactions parents give support and co-regulate the infant’s physiological, motoric and emotional arousal and activation. Through this process parents have a central role in supporting the infant’s development of self-regulation [17–19]. These patterns of early social interactions lay the foundation for the infant’s emotional attachment to their caregiver, with more optimal interactional patterns being predictive of a more secure attachment in the child [20, 21]. Furthermore, the quality of the parent-infant relationship and attachment is related to the child’s socio-emotional, cognitive and behavioural development [19, 22–25].

### The impact of parental stress and depression on the parent-infant relationship

Several studies have pointed to the close and complex relations between parenting stress, depression and maternal-infant bonding, and these factors have an important impact on the well-being of parents, infants and the parent-infant relationship [26–29]. Some level of stress or insecurity related to managing the new circumstances and the daily parental responsibilities in the postpartum period is common [2, 30]. Parenting stress is a broad term describing distress related to the demands of the parental role, and is the consequence of perceiving these demands as exceeding the available resources for coping [31]. Parenting stress can be negatively related to parental bonding [27], the quality of parenting and parent-child interactions [31, 32] and child developmental outcomes [32, 33].

Some mothers develop serious emotional distress during the postpartum period, and the prevalence rates for postpartum depression range from 10 to 15% [1, 34, 35]. Postpartum depression may negatively affect the parent-infant bond [28, 36] and interaction [37, 38]. It may also increase the risk for psychological or developmental difficulties in the child, including insecure attachment, internalising and externalising problems, and impaired social competence and language development [26, 37, 39].

### The newborn behavioral observation

The Newborn Behavioral Observation (NBO) is a relationship-based intervention delivered by NBO-trained health practitioners aiming to sensitise parents to the infant’s capacities, uniqueness and behavioural communication cues [5]. By increasing parental competence and confidence the intervention may contribute to more sensitive parenting and a positive parent-infant relationship. Previous studies have included first time mothers of healthy infants in the US [40–42] and Norway (qualitative study [43];), as well as mothers in risk of depression (Norwegian feasibility study [44];). The NBO has been delivered between 1 and 3 times in hospital and/or home settings. These studies suggest that the NBO can increase maternal engagement [40], sensitivity [41] and understanding of the child’s capacities and behavioural cues [40, 43, 44]. This may contribute to feeling more confident as a mother [43]. Results from a pilot-study indicated that receiving the NBO was associated with a reduced risk for depressive symptoms in first-time mothers [42].

### Aims

The present study reports data from mothers included in the Northern Babies Longitudinal Study (NorBaby [45, 46];). The aim was to evaluate the NBO as a universal preventive intervention within the regular well-baby clinic service by investigating the association between receiving the NBO and measures of depressive symptoms/parental stress and the mother-infant relationship in the first 4 months postpartum. We had three main hypotheses. First, based on the pilot-study by Nugent et al. [42], we hypothesised that receiving the NBO would be associated with lower levels of maternal depressive symptoms and parenting stress. Second, we hypothesised that the NBO would be associated with a stronger mother-infant relationship, measured as maternal-infant bonding, reflective functioning and confidence in the parenting role. Third, we hypothesised that the NBO would be associated with higher satisfaction/benefit of the postpartum follow-up.

## Methods

### Study design

The present study is part of a longitudinal study that had a non-randomised cluster-controlled design. Participants completed 6 measurement points (T) as follows: during gestational weeks 16–22 (T1), 24–30 (T2) and 31(T3), and at 6 weeks (T4), 4 months (T5) and 6 months (T6) after birth. However, after study commencement the interval for T1 was extended to increase recruitment, and the intervals for the other measurement points were extended due to delayed responses from participants. This article is based on data from T1, T4 and T5 (see Table 1 for study design). Participants completed T1 between gestational weeks 13 and 39 (median 23.0, mean 23.0, SD 3.62), T4 between 5 and 15 weeks after birth (median 7.6, mean 8.1, SD 1.94), and T5 between 3 and 9 months after birth (median 4.0, mean 4.4, SD 0.83). The time between completing T4 and T5 ranged between 5 and 28 weeks (median 13.0, mean 13.1, SD 3.62). The study design and procedure have been described in more detail earlier [45].

**Table 1:** Study design and outcome measures

Outcomes	T1 Pregnancy	Care as usual plus NBO vs care as usual	T4 ~ 6 weeks postpartum	T5 ~ 4 months postpartum	Description of outcomes
**Depression/stress**					
EPDS	•		•	•	Symptoms of depression
PSI^a^			•	•	Stress in the parenting role and parent-child relationship
**Mother-infant relationship**					
MPAS			•	•	The emotional bond from a mother towards her child
PRFQ			•	•	Mothers’ capacity to understand and reflect upon the child’s behaviours as expressions of underlying mental states
MCQ			•		Sense of competence in the parenting role
**Satisfaction/benefit with the postpartum follow-up**			•		How much mothers learned about the child’s signals and needs during follow-up. Satisfaction with the support and guidance received.

^a^The PSI-Parent domain was measured at T4, whereas both the PSI-Parent domain and the PSI-Child domain were measured at T5, *EPDS* Edinburgh Postnatal Depression Scale, *PSI* Parenting Stress Index, *PRFQ* Parental Reflective Functioning Questionnaire, *MPAS* Maternal Postnatal Attachment Scale, *MCQ* Maternal Confidence Questionnaire

Participants were allocated to the NBO intervention group or to care as usual based on their home address, which determined at which of five well-baby clinics they would receive their postpartum follow-up. Cluster randomisation of the well-baby clinics to learning the NBO was not feasible in this routine practice setting. Families belonging to one specific well-baby clinic received follow-up with the NBO plus care as usual. The NBO intervention was extended to three clinics during the study to increase the size of the NBO-group. Families at the remaining well-baby clinics received care as usual.

### Participants and procedure

All Norwegian-speaking pregnant women and their partners from Tromsø municipality in Northern-Norway were eligible for inclusion. The recruitment period was between October 2015 and December 2017. Pregnant women and partners attending the antenatal clinic were recruited by midwives who gave information about the study. Potential participants agreeing to be contacted were later telephoned by a member of the research team for more information about the study and to plan a meeting for inclusion. The final sample recruited was 220 women (approximately 12% of pregnant women in the region) and 130 of their partners. All participants gave written informed consent. Data was collected by means of online questionnaires answered during a meeting with a member of the research team (T1), or from home (T4, T5).

### The intervention

The NBO consists of 18 neurobehavioural observations focusing on the infant’s behavioural repertoire within the attentional-interactional, autonomic, motor and organisation of states domains [5]. This includes observations of responsivity to visual and auditory stimulation, capacity for habituation or sleep protection, amount of crying and ease of consoling, stress responses, reflexes, muscle tone and motor activity. Based on the observations, care giving strategies such as handling, sleep protection, comforting and regulation of social interaction are discussed [41]. The NBO takes 15 to 40 min to administer and can be used from birth until the infant is 3 months old. The observations are not performed as a checklist, but tailored to the needs of the individual family and the awake and sleep states of the infant [5]. Parents are encouraged to participate actively in the observation of their infant and to share their experiences, and the clinician meets them with a non-didactic and non-judgemental attitude. The overall aim is to provide tailored information and supervision related to parenting strategies based on the individual infant’s signals.

In this study, the NBO-group received three NBOs as an additional component to care as usual: 1. At the maternity ward with a midwife within 2 days post-delivery, 2. At the routine home visit with a public health nurse at 7–10 days post-delivery, and 3. NBO consultation at the well-baby clinic at 4 weeks post-delivery (additional to usual care visits). The comparison group received care as usual at the maternity ward, a home visit with a public health nurse at 7–10 days post-delivery, and had their first meeting at the well-baby clinic at 6 weeks post-delivery. Care as usual also included guidance on topics such as feeding, early social interaction, sleeping patterns, motor development, safe environment, crying, handling and caring for the baby, and the parents’ life situation and mental health [47]. In addition, the baby’s weight gain was evaluated. The NBO was integrated as part of the public health nurses’ regular practice. However, a distinction is that in the NBO the guidance is given as part of the observation of the baby and tailored to the unique baby’s state and behavioural communication cues, whereas care as usual may include more general guidance delivered as part of a conversation with the parents. The NBO was administered by certified midwives and public health nurses. They were instructed to keep logs after each NBO session to register the date of the NBO, who were present and which observation elements were performed.

### Research measures

Demographic information was collected at T1 and included questions about mental health history, physical health, education, work status before pregnancy, gross annual household income, marital status, number of previous children, social support from family and friends, and whether the pregnancy was wanted. The following four self-report questionnaires have been included for a description of sample characteristics and pre-intervention group differences. Pregnancy related anxiety (fear of giving birth, concerns about one’s appearance related to pregnancy, and fear of bearing a handicapped child) was measured with the 10-item Pregnancy-Related Anxiety Questionnaire-Revised (PRAQ-R [48];) at T1. Emotional, physical and sexual abuse, and household dysfunction during the parent’s own childhood and adolescence was measured with the 10-item questionnaire Adverse Childhood Experiences (ACE [49];) at T1. Depressive symptoms during the last 2 weeks was assessed with the 21-item Beck Depression Inventory-II (BDI-II [50];) at T1. The mother’s bonding towards her baby during pregnancy was measured with the 19-item Maternal Antenatal Attachment Scale (MAAS [51];) at T3 (between gestational weeks 31 and 41, median 34.0, mean 34.4, SD 2.23). For more information about these instruments see the study protocol [45]. See Table 1 for study design and a short description of the outcome measures.

#### Depression/stress measures

Symptoms of depression were assessed with the Edinburgh Postnatal Depression Scale (EPDS [52];). The EPDS is a 10-item self-report inventory using a 4-point Likert scale. The inventory assesses sadness, anxiousness, lack of enjoyment, self-blame and thoughts about self-harm. The 10 items yield a total score, and a study on a Norwegian sample suggests ≥10 as a cut off score for possible clinical depression [53]. The EPDS was included at both T1, T4 and T5. In the present study, EPDS had an acceptable to good internal consistency (Table 2).

**Table 2:** Cronbach’s alpha for the outcome measures

Outcomes	Cronbach’s alpha		
	Total	NBO-group	Comparison group
**Timepoint 4**			
EDPS	.79	.78	.80
PSI – PD	.92	.90	.92
PRFQ			
PM	.37	.32	.39
CMS	.74	.75	.73
IC	.69	.66	.71
MPAS	.81	.81	.82
MCQ	.81	.69	.85
**Timepoint 5**			
EPDS	.82	.84	.80
PSI – PD	.92	.91	.93
PSI – CD	.89	.85	.91
PRFQ			
PM	.41	.31	.50
CMS	.82	.80	.83
IC	.64	.60	.67
MPAS	.82	.81	.83

*EPDS* Edinburgh Postnatal Depression Scale, *PSI* Parenting Stress Index, *PD* Parent domain, *PRFQ* Parental Reflective Functioning Questionnaire, *PM* Pre-Mentalizing, *CMS* Certainty about Mental States, *IC* Interest and Curiosity, *MPAS* Maternal Postnatal Attachment Scale, *MCQ* Maternal Confidence Questionnaire, *CD* Child domain

Parenting stress was assessed with the Parenting Stress Index (PSI [54];). The PSI is a self-report inventory designed to assess stress in the parenting role, in the relationship between the parent and child and related to the perception of the child. The instrument consists of two domains: Parent domain (PD) and Child domain (CD). The PD was measured at T4 and T5, whereas the CD was measured only at T5. The PSI-PD consists of 54 items, divided into seven subdomains: competence, parent-infant bonding/attachment, isolation (e.g., social isolation and lack of social support), health (e.g., parental physical health), spouse (e.g., support from spouse), depression and role restriction. The PSI-CD consists of 47 items, divided into six subdomains: distractibility/hyperactivity, adaptability (e.g., the child’s ability to adapt to changes), parent reinforcement (e.g., experience of being liked by the child), demandingness, mood and acceptability (e.g., parental acceptance of the child). For the present study, we used the total scores from the PSI-PD and the PSI-CD. The Norwegian version of the PSI has been used in earlier research [55]. In the present sample, PSI had excellent internal consistency (Table 2).

#### Mother-infant relationship measures

Maternal bonding to the infant was measured at T4 and T5 with the Maternal Postnatal Attachment Scale (MPAS [14];). The MPAS is a self-report inventory consisting of 19 items, each with 2 to 5 response options, e.g., from “Very incompetent and lacking in confidence” to “Very competent and confident”. All items have a minimum and maximum score of 1 and 5, respective, and some items are reversed. The MPAS measures the mother’s pleasure in interacting with her baby, the mother’s level of irritation towards the baby, and the quality of the maternal bonding, e.g., feeling proud of the baby. The 19 items yield a total score, with higher scores indicating healthier bonding. The current version of MPAS was translated to Norwegian by members of the research team, under the consultation of a professional translator. In the present study, MPAS had good internal consistency (Table 2).

Maternal reflective functioning was assessed with the Parental Reflective Functioning Questionnaire (PRFQ [56];) at T4 and T5. PRFQ consists of 18 items, with response options on a 7-point Likert scale from “strongly disagree” (1) to “strongly agree” (7). The 18 items are equally divided into 3 subscales. The subscales measure different aspects of parental reflective functioning and are analysed separately in previous studies and not summed up to a total score [56]. The subscales are: Pre-Mentalizing (PM) modes (e.g., attributing negative intentions to the child and a lack of focus on the child’s inner life as a way of making sense of the child’s behaviour), Certainty about Mental States (CMS; parents’ ability to recognize that the child’s inner experiences are not always apparent), and Interest and Curiosity (IC) in mental states. Higher scores on all scales signals higher capacity for reflective functioning, whereas on the CMS subscale both high and low scores may be less optimal, indicating overconfidence or a too high degree of uncertainty in understanding the child’s states, respectively. The current version of the PRFQ was translated to Norwegian by A. Goksøyr and H. Braarud. In the present study, the PRFQ subscales had low to good internal consistency (Table 2).

Maternal confidence in parenting skills and the mother’s self-reported ability to perceive her child’s needs was assessed with the Maternal Confidence Questionnaire (MCQ [57];) at T4. The MCQ consists of 14 items, with response options on a 5-point Likert scale from “never” (1) to “very often” (5). Examples of items are: “I have all the skills need to be a good parent” and “When my baby is cranky, I know the reason”. Higher scores on the scale indicate a higher sense of competence. The Norwegian version of MCQ was used in earlier research [58]. In the present sample, MCQ had good internal consistency (Table 2).

#### Satisfaction/benefit of the postpartum follow-up measure

The mothers’ experiences of the professional follow-up after birth were assessed at T4 using questions developed for the present study. The first domain which consisted of five questions was: “Through the follow-up you have received after birth from the maternity ward and the well-baby clinic, how much have you learned about the child’s signals and needs in relation to:” (1) “the eating situation?”, (2) “Sleep/sleep patterns?”, (3) “Social interaction?”, (4) “Nappy change?”, and (5) “Crying/fuzziness?”. The second domain which consisted of four questions was: “In the follow-up you received after birth from the maternity ward and the well-baby clinic, how much did you feel you could:” (1) “Share thoughts and concerns?”, (2)“Ask questions”, (3) “Get practical guidance?”, and (4) “Have trust in the health care worker?”. The last domain was: “Overall, to what extent do you feel that the follow-up has supported you and your family in a satisfactory manner?” Participants answered the questions on 5-point Likert scales from 1 (“Nothing”/“To a very small extent”) to 5 (“Very much”/“To a very large extent”). These questions are treated as single items and Cronbach’s alphas are therefore not reported.

### Statistical analysis

All statistical analyses were carried out using IBM SPSS Statistics for Windows, Version 25.0 [59]. Analysis of missing values revealed that all dependent variables contained missing data with a total missing value frequency of 22.1% and with 97 participants providing data for all measures at each meeting. The majority of participants (40 cases, 18.2%) had only one missing value, whereas 24 cases (10.9%) missed at least 80% of our dependent variables. By excluding these 24 participants, the final analysis sample consisted of 196 participants (NBO-group: *N* = 82; comparison group: *N* = 114), with all providing data for each questionnaire on at least one of the post-NBO meetings. In our final sample, 8 participants missed data collection at T4 (NBO-group: *N* = 6), while data from T5 was missing in 26 cases (NBO-group: *N* = 9). That is, 82.7% participated on both T4 and T5, even though some did not complete all scales or items. Moreover, data from the PSI was missing in most participants (PSI-PD: *N* = 52, PSI-CD: *N* = 46), but even here, the proportion of missing data from both groups were comparable (PSI-PD: NBO-group: *N* = 22 (26.8%), comparison group: *N* = 30 (26.3%); PSI-CD: NBO-group: *N* = 18 (21.9%), comparison group: *N* = 28 (24.5%)). Finally, data from the EPDS-T4, MCQ-T4 and PRFQ-T4 was collected in most cases (*N* = 184, NBO-group: *N* = 76 (92.6%), comparison group: *N* = 108 (94.7%)). We replaced missing data using the multiple imputation method with 50 iterations, by including data available from all scales and questionnaires as both predictors and predicted variables. Comparisons of participants missing the dependent variables (*N* = 24) with the final sample (*N* = 196) showed significant differences with regard to age, education, work status before pregnancy and annual household income. Missing participants were younger (*p* = .007, 29.0 vs 31.6 years), had a lower level of education (*p* < .001), lower income (*p* = .018) and were less often in full-time work before pregnancy and more often students (*p* = .003). There were no significant differences between the final sample and the excluded group on any other demographic or clinical variables.

By comparing basic demographic variables (e.g., age, well-baby clinic, level of education, gross annual household income, number of previous children, whether the current pregnancy was wanted, presence of any physical health problems, and previous occurrence of depressive symptoms, see Table 3 for all variables), a significant effect of group membership (comparison group vs. NBO) was found for the level of education only (Z = − 2.19, *p* = .028), since the number of participants with 4 or more years spent in higher education was nearly twice in the comparison relative to the NBO group (74 vs. 41 participants, respectively). All other demographic parameters were comparable between the two groups (*p’s* > .08 for all). Similarly, potential group differences in any of our baseline measures (BDI-II at T1, PRAQ at T1 and T3, ACE at T1, MAAS at T3) collected prior to the intervention were assessed using independent-samples *t*-tests, revealing no significant effect of group (*p’s* > .19). Therefore, we added the level of education (3 levels: upper secondary school or lower, < 4 years or ≥ 4 years in higher education) as a covariate for all statistical analyses to control for the potential contribution of education to the observed effects. Given the cluster-controlled design of our study [45], we also included well-baby clinic as another covariate, coded as a dummy variable.

**Table 3:** Description of demographic and clinical variables at inclusion for the groups receiving the Newborn Behavioral Observation (NBO) and care as usual (*n* = 196)

Variable	NBO (***n*** = 82)	Comparison group (***n*** = 114)	Test statistic (***t*** or ***χ***^***2***^)	***p***-value	Effect size (Cohen’s ***D*** or Cramer’s ***V***)
**Mean age in years (SD)**^**a**^	31.00 (3.96)	31.14 (4.46)	0.23	.82	.033
**Marital status, n (%)**					
Married or cohabiting	81 (98.8)	109 (95.6)	1.61	.20	.233
**Education, n (%)**^**a**^			4.84	.08	.701
Upper secondary school or less	12 (14.6)	10 (8.8)			
< 4 years higher education	29 (35.4)	29 (25.4)			
≥ 4 years higher education	41 (50.0)	74 (64.9)			
**Work status before pregnancy**^**a**^			5.04	.41	
Full-time	70 (85.4)	99 (86.8)			
Part-time	3 (3.7)	0			
Student	7 (8.5)	10 (8.8)			
Homemaker	0	1 (0.9)			
Unemployed	1 (1.2)	1 (0.9)			
Sick leave or disability benefits	1 (1.2)	2 (1.8)			
**Family income, n (%)**^**b**^			2.32	.31	
≤ 350,000 NOK (38,799 USD)	6 (7.3)	3 (2.6)			
351,000–750,000 NOK (38,910–83,141 USD)	21 (25.6)	29 (25.4)			
≥ 751,000 NOK (83,252 USD)	55 (67.1)	80 (70.2)			
**Wanted pregnancy, n (%)**^**d**^	79 (96.3)	108 (94.7)	0.28	.59	
**Parenting experience, n (%)**			0.45	.80	
First-time mother	38 (46.3)	57 (50.0)			
Second-time mother	37 (45.1)	46 (40.4)			
Two or more previous children	7 (8.5)	11 (9.6)			
**Mental health, n (%)**					
Lifetime mental health problems	28 (34.1)	40 (35.1)	0.02	.89	.003
Previous depressive symptoms^e^	28 (34.1)	36 (31.6)	0.14	.70	.02
Contact with mental health services	24 (29.3)	36 (31.6)	0.12	.73	.017
**Physical health, n (%)**			1.10	.58	
Pregnancy-related physical health problems^a^	25 (30.5)	37 (32.5)	0.08	.77	
Other physical health problems^c^	8 (9.8)	16 (14.0)	0.81	.37	
**Social support, n (%)**					
Family can help when in need	74 (90.2)	109 (95.6)	2.22	.14	.322
Friends can help when in need	72 (87.8)	104 (91.2)	0.61	.43	.088
Can confide in family	73 (89.0)	95 (83.3)	1.26	.26	.182
Can confide in friends	75 (91.5)	109 (95.6)	1.43	.23	.207
**Clinical questionnaires, M (SD)**					
Edinburgh Postnatal Depression Scale	4.30 (3.09)	4.66 (3.81)	0.50	.61	.072
Beck Depression Inventory-II	7.79 (4.15)	7.98 (5.89)	0.20	.84	.029
Pregnancy-Related Anxiety Questionnaire	23.26 (7.55)	23.55 (8.34)	0.25	.80	.036
Adverse Childhood Experiences	0.79 (1.37)	0.98 (1.67)	0.75	.45	.109
Maternal Antenatal Attachment Scale	73.63 (7.67)	74.86 (6.88)	0.56	.57	.081

Missing data: ^a^*n* = 1, ^b^*n* = 2, ^c^*n* = 3, ^d^*n* = 4, ^e^Previous experience with being depressed most of the day, almost each day for a period of two weeks

**Table 4:** Means, standard deviations and range for all outcome measures for the NBO-group (*n* = 82) and comparison group (*n* = 114). For the PRFQ means, standard deviations and range is calculated per item

Outcomes	NBO (*n* = 82)		Comparison group (*n* = 114)		***t***-value	***p***-value	Effect size
	M (SD)	Range	M (SD)	Range			
**Timepoint 4**							
EDPS	3.61 (3.12)	0–14.00	3.55 (3.19)	0–13.00	0.41	.68	.059
PSI – PD	115.91 (18.56)	73.00–158.00	113.56 (21.89)	71.46–193.00	0.67	.50	.097
PRFQ							
PM	6.66 (0.39)	5.17–7.12	6.54 (0.49)	5.17–7.00	1.72	.09	.249
CMS	4.11 (0.88)	1.83–6.33	4.03 (0.91)	1.67–6.00	0.81	.42	.117
IC	5.88 (0.80)	2.67–7.00	6.02 (0.83)	2.67–7.00	−0.97	.33	−.14
MPAS	82.26 (7.27)	58.50–94.00	82.18 (7.68)	46.40–95.00	0.04	.97	−.006
MCQ	59.19 (4.03)	47.00–67.00	59.33 (5.75)	36.00–70.00	−0.08	.93	.012
**Timepoint 5**							
EPDS	3.15 (3.41)	−0.14 - 15.00	3.60 (3.44)	−0.82 - 14.00	−1.23	.22	−.178
PSI – PD	114.04 (20.87)	73.45–164.00	113.90 (23.52)	68.92–189.00	0.05	.96	.007
PSI – CD	84.00 (12.63)	57.00–123.00	86.51 (16.75)	53.00–159.00	− 0.41	.68	−.059
PRFQ							
PM	6.66 (0.37)	5.50–7.00	6.71 (0.38)	4.67–7.09	−0.83	.41	−.12
CMS	4.26 (0.97)	1.33–6.00	4.36 (1.01)	1.50–6.67	−0.72	.47	−.104
IC	6.19 (0.64)	4.00–7.00	6.08 (0.73)	3.17–7.00	0.84	.40	.122
MPAS	84.21 (6.46)	64.70–93.60	83.04 (7.67)	42.30–95.41	0.88	.38	.127

*EPDS* Edinburgh Postnatal Depression Scale, *PSI* Parenting Stress Index, *PD* Parent domain, *PRFQ* Parental Reflective Functioning Questionnaire, *PM* Pre-Mentalizing, *CMS* Certainty about Mental States, *IC* Interest and Curiosity, *MPAS* Maternal Postnatal Attachment Scale, *MCQ* Maternal Confidence Questionnaire, *CD* Child domain

To assess the differences between the NBO-group and the comparison group, the sample size for our study was based on a priori power analysis using multivariate analysis of variance (MANOVA) with an estimated effect size of f^2^ = 0.07, a power of 0.8 and an alpha level of .05 [45]. This approach enables evaluating the joint effect of the intervention on psychometric scales sensitive to overlapping psychological constructs, accounting for possible covariations between them. We conducted separate MANCOVAs (Multivariate analysis of covariance) for testing the differences between the two groups on our two domains of interest: depressive symptoms/parental stress and mother-infant relationship, while controlling for the level of education and well-baby clinic. Separate analyses were performed at time points T4 and T5 (at ~week 6 and ~months 4 postpartum, respectively). We used scores from the EPDS, PSI-PD and PSI-CD for estimating maternal depression/stress (with PSI-CD being available at T5 only), whereas subscales from the PRFQ (PRFQ-PM, PRFQ-CMS, PRFQ-IC), the MPAS and the MCQ were used to assess the mother-infant relationship. Even though some variables showed signs of skewness and/or kurtosis (i.e., values > 1 or < − 1) indicative of non-normal distributions, given the relatively large sample size of the current study and that assumptions of homogeneity of variances and covariance matrices were not violated (with the only exception of the PRFQ-PM score collected at T4 with a significant Levene’s test of *F*(1,193) = 7.45, *p* = .007), we decided to proceed with our original multivariate approach with reporting Pillai’s trace statistics (*V*) and calculating 95% bias-corrected and accelerated (BCa) bootstrapped confidence intervals for the contribution of NBO status to each outcome variable [60]. For both domains of interest, separate analyses were performed at time points T4 and T5, with scores from the MCQ being available at T4 only. In order to investigate if the differences between the two groups were changing from T4 to T5, scores for the two domains were entered into repeated-measures ANCOVAs (Analysis of covariance) with Time (T4, T5) and Questionnaire (maternal depression/stress: EPDS, PSI; mother-infant relationship: PRFQ, MPAS) as within-subject variables, NBO-group as the between-subject variable, and Education and well-baby clinic as covariates.

Finally, responses to the questions regarding satisfaction with the follow-up from both groups were compared using Mann-Whitney *U* test. Potential variations in follow-up responses across the six well-baby clinics of intervention were assessed separately for the NBO and comparison groups with Kruskal-Wallis test. All statistical analyses were performed with an alpha value of .05, with Bonferroni correction applied for follow-up ANOVAs evaluating the contribution of each dependent variable to the joint effects revealed by the MANCOVA approach. Effect size is reported using Cohen’s *f*^*2*^.

## Results

Demographic and clinical data for the two groups at inclusion/pre-intervention is reported in Table 3. Mothers in both groups had a mean age of approximately 31 years and above 95% were married or cohabiting. About half of the women in both groups were first-time mothers and a vast majority reported wanting this pregnancy. A substantial proportion of both groups had more than 4 years of higher education and reported having a gross annual family income above 750,000 NOK (~ 83,252 $), and above 85% in both groups were working full-time before pregnancy. About one third in both groups reported lifetime mental health problems, previous depressive symptoms and having been in contact with mental health services at some point during their life. However, participants in both groups scored well below the clinical threshold on the EPDS at inclusion (Table 3). For a description of the full sample (*n* = 220) see Supplementary Table 1. Scores on all outcome measures for both groups can be found in Table 4.

### Exposure to the intervention

The NBO-logs filled out by midwives and public health nurses after each NBO session indicated that NBO was delivered to 64.1% of mothers in the NBO group at the maternity ward, 71.7% at the home visit, and 69.6% at the well-baby clinic at 4 weeks after birth. Thus, 7.6% of mothers in the intervention group received 1 session with NBO, 33.7% received 2 sessions, and 43.5% received 3 sessions. This means that at least 84.8% of the mothers in the intervention group received at least one NBO session, and thus, were exposed to the intervention content. The logs indicated that 15.2% did not receive the intervention at all. However, communication with the maternity ward and well-baby clinics delivering the NBO indicated that it took some time before filling out the logs became part of the routine. Therefore, the reported numbers should be interpreted as conservative estimates, as it is likely that NBO sessions were performed without the health care worker keeping a log.

### Maternal depressive symptoms/parental stress

Testing for the difference between the NBO- and the comparison group on depressive symptoms/parental stress, we did not find significant differences at any time point in the multivariate analysis (at T4: *V* = 0.015, *F*(2,186) = 1.41, *p* = .246, *f*^*2*^ = .015; at T5: *V* = 0.027, *F*(3,185) = 1.69, *p* = .170, *f*^*2*^ = .027). Moreover, we found no significant effects of group membership for any questionnaire score when tested separately at each time point (EPDS-T4: *F*(1,187) = 1.48, *p* = .225, *f*^*2*^ = .008; PSI-PD-T4: *F*(1,187) = 2.78, *p* = .094, *f*^*2*^ = .015; EPDS-T5: *F*(1,187) < 0.01, *p* = .985, *f*^*2*^ < .001; PSI-PD-T5: *F*(1,187) = 2.34, *p* = .128, *f*^*2*^ = .012; PSI-CD-T5: *F*(1,187) = 0.11, *p* = .736, *f*^*2*^ = .001). However, we found a significant main effect of Education (at T4: *V* = 0.06, *F*(2,186) = 5.56, *p* = .004, *f*^*2*^ = .06; at T5: *V* = 0.08, *F*(3,185) = 5.37, *p* = .001, *f*^*2*^ = .09), such that participants with the highest education level (≥ 4 years in higher education) scored lower on the EPDS but not on the PSI at T4 and T5 (EPDS-T4: *F*(1,187) = 10.84, *p* = .001, *f*^*2*^ = .06, *b* = − 1.06, BCa 95% CI [− 1.70, − 0.42]; EPDS-T5: *F*(1,187) = 11.27, *p* = .001, *f*^*2*^ = .06, *b* = − 1.20, BCa 95% CI [− 2.00, − 0.39]; PSI-PD-T4: *F*(1,187) = 2.44, *p* = .120, *f*^*2*^ = .01, *b* = − 3.41, BCa 95% CI [− 7.44, 0.78]; PSI-PD-T5: *F*(1,187) = 1.23, *p* = .269, *f*^*2*^ = .01, *b* = − 2.58, BCa 95% CI [− 7.26, 1.86]; PSI-CD-T5: *F*(1,187) = 0.74, *p* = .390, *f*^*2*^ = .004, *b* = 1.31, BCa 95% CI [− 1.72, 4.16]).

We also performed a repeated-measures ANCOVA with EPDS and PSI scores collected at T4 and T5 as predictors. We did not observe a significant main effect or interactions for group-allocation (NBO vs. comparison group; *F*’s < 2.92, *p*’s > .088, *f*^*2*^’s < .016). However, after controlling for EPDS scores collected at baseline (T1), both the main effect for NBO group (*F*(1,186) = 4.61, *p* = .033, *f*^*2*^ = .02) and the interaction between NBO and Questionnaire were significant (*F*(1,186) = 4.49, *p* = .035, *f*^*2*^ = .02), with the latter indicating comparable EPDS values between the two groups (*p* = .172), but higher PSI scores in the NBO group (*p* = .033, Bonferroni-corrected). However, these effects were not significant when including only first-time mothers in the analysis (*F*’s < 3.61, *p*’s > .060, *f*^*2*^’s < .041).

Finally, we investigated the proportion of participants in the two groups who scored above a cutoff of 10 on the EPDS, indicating possible clinical depression [53]. At T4, this included 4.9% (*n* = 4) and 6.1% (*n* = 7) of the NBO- and comparison group, respectively. The numbers at T5 were 4.9% (*n* = 4) and 4.4% (*n* = 5), respectively. The differences between the groups were not significant at either time point (T4: Fisher’s exact: *p* = .76, T5: Fisher’s exact: *p* = 1.00).

### Mother-infant relationship measures

As for the mother-infant relationship domain (PRFQ-PM, PRFQ-CMS, PRFQ-IC, MPAS and MCQ), there was no significant main effect for NBO at any time point (at T4: *V* = 0.04, *F*(5,183) = 1.56, *p* = .171, *f*^*2*^ = .04; at T5: *V* = 0.03, *F*(4,184) = 1.25, *p* = .289, *f*^*2*^ = .03). Testing the effect of NBO separately for each questionnaire and each time point did not yield significant results either (PRFQ-PM-T4: *F*(1,187) = 0.43, *p* = .513, *f*^*2*^ = .002; PRFQ-CMS-T4: *F*(1,187) = 1.58, *p* = .210, *f*^*2*^ = .008; PRFQ-IC-T4: *F*(1,187) = 0.46, *p* = .500, *f*^*2*^ = .002; MPAS-T4: *F*(1,187) = 2.11, *p* = .148, *f*^*2*^ = .011; MCQ-T4: *F*(1,187) = 0.17, *p* = .681, *f*^*2*^ = .001; PRFQ-PM-T5: *F*(1,187) = 1.52, *p* = .219, *f*^*2*^ = .008; PRFQ-CMS-T5: *F*(1,187) = 0.36, *p* = .550, *f*^*2*^ = .002; PRFQ-IC-T5: *F*(1,187) = 1.19, *p* = .276, *f*^*2*^ = .006; MPAS-T5: *F*(1,187) = 0.97, *p* = .325, *f*^*2*^ = .005). Repeated-measures ANCOVA investigating between-group differences in changes in PRFQ and MPAS scores over time (from T4 to T5) indicated no significant main effect for the NBO group (*F*(1,187) = 0.28, *p* = .598, *f*^*2*^ = 0.001), but a significant three-way Time x NBO x Questionnaire interaction (*F*(3,561) = 3.92, Greenhouse-Geisser *ε* = .9, *p* = .011, *f*^*2*^ = .02). However, Bonferroni-corrected post hoc comparisons revealed no significant differences between the two groups for any questionnaire score at any time point (*p*’s > .147).

### Satisfaction/benefit of the postpartum follow-up

Finally, we compared responses to our follow-up questions at T4 (mean 8.1 weeks postpartum) asking either about how much they learned during the previous consultations with the healthcare professionals about the child’s signals and needs in everyday situations (i.e. eating, sleep/sleep patterns, social interaction, nappy change, crying/fuzziness) or inquiring about their feelings related to the interaction with healthcare professionals and their satisfaction with the meetings. Here we found significantly higher scores indicative of more efficient interventions for the NBO group for questions related to sleep/sleep patterns (Z = -2.98, *p* = .003), social interaction (Z = -2.79, *p* = .005) and crying/fuzziness (Z = -3.93, *p* < .001). We did not find differences in the distribution of responses between the well-baby clinics for either group of participants (NBO: H(2) < 4.3, *p* > .11; comparison group: H(2) < 5.12, *p* > .27).

### Exploratory analysis including only first-time mothers

As an exploratory analysis, we conducted the above tests by including only first-time mothers, as we considered the possibility that NBO would lead to more beneficial outcomes for this group (due to the lack of previous experience with being a mother). This subsample consisted of 59 participants without NBO and 36 participants allocated to the intervention-group. However, this did not change the above results. The only exception was that these analyses failed to find significant group differences for any of the questions regarding satisfaction/benefit of the follow-up, with the item asking about social interaction showing a trend only (Z = -1.90, *p* = .057). This was possibly due to the highly reduced power of these exploratory analyses.

## Discussion

The present study evaluated the association between postpartum follow-up with the NBO and a broad range of measures related to maternal depressive symptoms, stress, the mother-infant relationship and satisfaction/benefit of the postpartum follow-up. The results confirmed only one of our original hypothesis as they indicated that the NBO-group reported significantly higher benefit of the postpartum follow-up compared to the comparison group. Specifically, they learned significantly more from the follow-up about the baby’s signals and needs in relation to sleep/sleep patterns, social interaction and crying/fuzziness. Our hypothesis that the NBO would be associated with lower levels of depressive symptoms and parenting stress and higher scores on assessments related to the mother-infant relationship were not confirmed. For the mother-infant relationship domain neither the MANCOVAs, nor the repeated measures ANCOVA did show significant benefit for the NBO-group for either time point. Also, on the depressive symptoms/parental stress domain there were few differences between the groups. However, in the repeated measures ANCOVA we found a numerically small, but significant difference between the groups on the PSI Parent Domain, with the participants in the NBO-group indicating slightly higher parental stress.

The most clear-cut differences between the groups were found for questions regarding the participants’ experience of the postpartum follow-up, specifically, how much they learned about the baby’s signals and needs. These questions most directly tap into how participants experienced the NBO. The significant differences between the groups for the areas of sleep/sleep patterns, social interaction and crying/fussiness correspond well with the content of the intervention [5]. Social interaction is an overarching focus of the NBO and several observational elements focus on this. In addition, the intervention also includes specific elements concerning sleep/sleep protection and strategies for supporting and comforting the baby. These results correspond well with the results from another Norwegian study of the feasibility and acceptability of the NBO showing that parents rated the intervention as highly useful with regard to understanding the behavioural cues of their infants [44]. It should be mentioned that the comparison group also received follow-up by skilled health care professionals and provided as high ratings as the NBO-group on general satisfaction with the follow-up, the practical guidance and the relationship with the health care worker. Despite this strong comparator, the NBO-group rated important parts of the follow-up significantly higher. Compared to general guidance a key feature of the NBO is that it provides guidance that is tailored to the observations of the unique baby’s state and communication cues. The present result emphasizes that using the NBO clearly provides parents with a better grasp of the baby’s signals and needs in important everyday situations. This could over time, potentially have extended effects on other important parental outcomes such as sensitivity, and child developmental outcomes. However, although parents rated the intervention as useful, we do not have information about the actual sleep patterns of the infants or observational measures of parental behaviours and parent-infant interaction.

The results from the mother-infant relationship domain did not support our hypothesis that the NBO would be associated with higher levels of maternal bonding, reflective functioning and confidence. Thus, our results were not in line with earlier research indicating that the NBO can significantly increase maternal engagement [40], confidence [43] and maternal sensitivity [41]. However, the present study did not include any observational measures of actual mother-infant interaction, which could possibly have shed additional light on this issue.

Also, the lack of differences between the groups on the depressive symptoms/parental stress domain was contrary to our hypothesis. We found no main effect of the NBO when looking at the overall depressive symptoms/parental stress domain comprising of both the EPDS and the PSI– Parent domain at T4 and T5 using MANCOVAs. Nor did we find an effect of the NBO when investigating potential differences between groups in number of participants scoring above cut-off for probable clinical depression. Hence, our findings contradicts the results of a previous pilot-study [42] which indicated that the use of NBO may be associated with a substantial reduction in the risk of major depression. In the repeated-measures ANCOVA we found significantly higher PSI Parent Domain scores for the NBO-group. However, the mean difference between the groups was numerically very small (< 2.5 points) and difficult to interpret as both groups scored low on parenting stress compared to women with postpartum depression [61]. Further studies are needed to evaluate if this is a reliable effect of the NBO or a spurious finding.

Overall, the present study found limited benefits for the NBO for both the mother-infant relationship domain and the depressive symptoms/parental stress domain. This could possibly be due to the generally well-functioning sample, based on their educational level, social support, pre-pregnancy work status and income. The mean level of depressive symptoms was low at all measurement points, and the level of maternal stress was low compared to women with postpartum depression [61]. Scores on the MPAS and MCQ were also high for both groups indicating high levels of mother-infant bonding and maternal confidence. There is a possibility that the high functioning of this sample did not give much room for significant enhancement by receiving the NBO, neither on the depressive symptoms/parental stress domain nor on the mother-infant relationship domain. Two previous studies of NBO conducted in the US with a more limited sample size have indicated that the intervention may have beneficial effects by reducing the odds of depression in first-time mothers [42] and increasing sensitivity in mother- infant interactions [41]. Contrary to this, our results suggest that the benefits of the NBO on depressive symptoms, parenting stress and measures related to the mother-infant relationship may be limited within a general population sample with particularly well-functioning participants. Similar results were found in a meta-analysis showing that interventions to enhance parental sensitivity were in general more effective in clinical samples compared to non-clinical samples [62]. Furthermore, a recent study of a video-based intervention to improve the parent-infant relationship did not find significant effects of the intervention for well-functioning parent-child dyads [63], despite positive effects for similar interventions within risk families [64]. In line with this, the NBO may possibly have more significant effects on depressive symptoms, parenting stress and mother-infant relationship measures within a risk population. In addition, as previously mentioned, also the comparison group received close follow-up focus in on similar topics as the NBO from well-trained health professionals. The high quality of the usual care is also supported by the high satisfaction with postpartum follow-up reported by the comparison group. This makes it even more challenging to reveal significant intervention effects in a largely well-functioning sample. Lastly, as shown by the intervention logs, although the majority of participants in the NBO-group received one or more sessions, not all participants received the full 3-session intervention and 15% did not receive any sessions. This may have reduced group differences.

### Strengths and limitations

The present study has some strengths. The study is a longitudinal study following participants from pregnancy until the baby was 6 months old. Participants completed comprehensive measures on important demographic and clinical variables, as well as important variables related to the parent-child relationship and parental functioning. The outcomes were assessed using standardised, reliable and validated measures developed for use with the current population [14, 54, 56, 65–67]. Another strength is that the intervention was delivered within regular practice as part of the routine postpartum follow. The study also has several limitations. Unfortunately, individual or cluster randomisation of participants to the NBO and comparison group was not feasible within this routine practice setting. However, the only group difference at inclusion was on educational level, where the intervention group had a slightly lower educational level than the control group. Controlling for this variable in the analyses did not make any difference to the results. Potentially there could be differences between participants belonging to different well-baby clinics. However, controlling for this variable did not affect the results. Another limitation is that far from all families received all three NBO sessions. An additional limitiation with the design is that the NBO-group received one additional follow-up session compared to the comparison group (NBO at 4 weeks postpartum). This adds some uncertainty to the results as it cannot be ruled out a dose-response effect of the NBO or that any benefits of the NBO could be related to receiving more follow-up from a health professional rather than to features of the intervention itself. Other limitations concern the measurements. Although well-validated questionnaires were chosen all variables included in this study were measured using self-reports. Including interviews could have provided for instance more reliable diagnostic information. In addition, it was impossible to collect pre-measures filled-out before the intervention for several of the outcome variables. This is due to the fact that these measures focus on the experience of being a parent, and thus cannot be answered before the baby is born. As the first NBO-session was delivered already within 2 days post-delivery, there was no time to include pre-measures for the parental questionnaires. However, for the EPDS we did control for depressive symptoms before birth. Another limitation concerns the low internal consistency of the PRFQ subscale pre-mentalizing for both time points. Earlier research has found acceptable internal consistency for this subscale [56], but also internal consistency in the low range [68]. One possibility for the low reliability in our data may be that some of the questions were difficult for parents to answer at this early stage of infancy since the scale is designed for parents of children up to several years old [56]. Additionally, for some of the PRFQ-items the socially acceptable answers might be quite easy to pick up by the participants. Lastly, the sample was generally well-functioning. Most participants were married or cohabiting with high levels of perceived social support and a large proportion had higher education, were working full-time before pregnancy and had a quite high family income. The mean level of depressive symptoms was low at all time points, despite about a third of the sample reporting previous mental health problems. Due to the generally high socioeconomic status of the sample, the generalisability of the results to other less advantaged populations is uncertain.

## Conclusion

Although, the associations between the NBO, maternal mental health and relationship measures were scarce, the results imply that participants from the NBO-group learned more than the comparison group about reading their infant’s signals in important everyday situations related to social interaction, sleep/sleep patterns and crying/fuzzyness. Our results suggest that the benefits of the NBO may be limited within a general population sample of particularly well-functioning participants. However, more research is needed to investigate if the effects of the NBO could be stronger on other outcome measures, such as measures of child development, parent-infant interaction and child attachment classification, or with more disadvantaged populations. There is also a possibility that the effects from the NBO could be more evident later on, as is evident from other studies using different interventions [69]. We therefore encourage future studies to explore the effects of the NBO within a longer time frame.

## Supplementary information

**Additional file 1: Supplementary Table 1.** Description of demographic and clinical variables at inclusion for the groups receiving the Newborn Behavioral Observation and care as usual (full sample, *n* = 220).

## Data Availability

The dataset used during the study is available from the corresponding author on request.

## Abbreviations

*ACE*: Adverse Childhood Experiences
*ANCOVA*: Analysis of covariance
*BDI-II*: Beck Depression Inventory-II
*EPDS*: Edinburgh Postnatal Depression Scale
*MAAS*: Maternal Antenatal Attachment Scale
*MANOVA*: Multivariate analysis of variance
*MANCOVA*: Multivariate analysis of covariance
*MCQ*: Maternal Confidence Questionnaire
*MPAS*: Maternal Postnatal Attachment Scale
*NBO*: Newborn Behavioral Observation
*PRAQ*: Pregnancy-Related Anxiety Questionnaire-Revised
*PRFQ*: Parental Reflective Functioning Questionnaire
*PRFQ-PM*: Parental Reflective Functioning Questionnaire-Pre Mentalizing
*PRFQ-CMS*: Parental Reflective Functioning Questionnaire-Certainty about Mental States
*PRFQ-IC*: Parental Reflective Functioning Questionnaire-Interest and Curiosity
*PSI*: Parenting Stress Index
*PSI-PD*: Parenting Stress Index-Parent domain
*PSI-CD*: Parenting Stress Index-Child domain
