# Supplementary material for: Newborn Behavioral Observation, maternal stress, depressive symptoms and the mother-infant relationship: results from the Northern Babies Longitudinal Study (NorBaby)
Source: BMC Psychiatry. 2020 Jun 15;20:300. doi: 10.1186/s12888-020-02669-y (PMC7294655; doi:10.1186/s12888-020-02669-y)
Supplement: Supplementary file 1 — Additional file 1: Supplementary Table 1. Description of demographic and clinical variables at inclusion for the groups receiving the Newborn Behavioral Observation and care as usual (full sample, n = 220). [file 12888_2020_2669_MOESM1_ESM.docx]

**Supplementary table 1.** Description of demographic and clinical variables at inclusion for the groups receiving the Newborn Behavioral Observation and care as usual (full sample, n = 220).

| **Variable** | **NBO**  **(n = 91)** | **Comparison group (n = 129)** | **Test statistics** |
| --- | --- | --- | --- |
| **Mean age in years (SD)^a^** | 30.7 (4.02) | 30.8 (4.71) | *t*(211.55) = 0.20. *p* = .84 |
| **Marital status, n (%)^a^** |  |  | Fisher’s exact: *p* = .24 |
| Married or cohabiting | 90 (98.9) | 123 (95.3) |  |
| Single | 1 (1.1) | 6 (4.7) |  |
| **Education, n ( %)^b^** |  |  | Χ^2^ (2) = 4.12, *p* = .13 |
| High school or less | 15 (16.3) | 17 (13.2) |  |
| ≤ 4 years higher education | 33 (35.9) | 33 (25.6) |  |
| > 4 years higher education | 43 (46.7) | 78 (60.5) |  |
| **Work status before pregnancy^b^** |  |  | Fisher’s exact = 2.67, *p* = .86 |
| Full-time | 75 (81.5) | 108 (83.7) |  |
| Part-time | 4 (4.3) | 2 (1.6) |  |
| Student | 9 (9.8) | 13 (10.1) |  |
| Homemaker | 0 | 1 (0.8) |  |
| Unemployed | 1 (1.1) | 1 (0.8) |  |
| Sick leave or disability benefits | 2 (2.2) | 3 (2.3) |  |
| **Family income, n (%)^c^** |  |  | Χ^2^ (2) = 1.39, *p* = .50 |
| ≤ 350 000 NOK | 6 (6.5) | 5 (3.9) |  |
| 350 000 – 750 000 NOK | 28 (30.4) | 34 (26.4) |  |
| ≥ 750 000 NOK | 57 (62.0) | 88 (68.2) |  |
| **Wanted pregnancy, n (%)^e^** | 88 (95.7) | 121 (93.8) | Fisher’s exact = 2.70, *p* = .29 |
| **Parenting experience, n (%)** |  |  | *t*(218) = 0.15. *p* = .84 |
| First-time mother | 44 (48.4) | 64 (49.6) |  |
| Second-time mother | 39 (42.9) | 52 (40.3) |  |
| Two or more previous children | 8 (8.8) | 13 (10.1) |  |
| **Mental health, n (%)^a^** |  |  |  |
| Lifetime mental health problems | 31 (33.7) | 46 (35.7) | Χ^2^ (1) = 0.60, *p* = .89 |
| Previous depressive symptoms^h^ | 31 (33.7) | 41 (31.8) | Χ^2^ (1) = 0.13, *p* = .72 |
| Contact with mental health services | 28 (30.4) | 42 (32.6) | Χ^2^ (1) = 0.08. *p* = .78 |
| **Physical health, n (%)** |  |  |  |
| Pregnancy-related physical health problems^a^ | 32 (35.2) | 41 (31.8) | X^2^ (1) = 0.24, *p* = .63 |
| Other physical health problems^c^ | 11 (12.1) | 18 (14.0) | X^2^ (1) = 0.13, *p* = .72 |
| **Social support, n (%)^a^** |  |  |  |
| Family can help when in need | 82 (89.1) | 122 (94.6) | Χ^2^ (1) = 1.58, *p* = .21 |
| Friends can help when in need | 81 (88.0) | 117 (90.7) | Χ^2^ (1) = 0.17, *p* = .68 |
| Can confide in familiy | 82 (89.1) | 106 (82.2) | Χ^2^ (1) = 2.71, *p* = .10 |
| Can confide in friends | 84 (91.3) | 123 (95.3) | Χ^2^ (1) = 0.89, *p* = .35 |
| **Clinical questionnaires, M (SD)** |  |  |  |
| Edinburgh Postnatal Depression Scale^d^ | 4.4 (3.2) | 4.7 (3.7) | *t*(214) = -0.63. *p* = .53 |
| Pregnancy-Related Anxiety Questionnaire^g^ | 23.1 (7.6) | 23.6 (8.5) | *t*(207) = -0.41. *p* = .68 |
| Adverce Childhood Experiences^f^ | 0.8 (1.4) | 1.0 (1.7) | *t*(209) = -0.88. *p* = .38 |

Note. Missing data: ^a^n = 1, ^b^n =2, ^c^n = 3, ^d^n = 4, ^e^n = 5, ^f^n = 9, ^g^n = 11; ^h^Previous experience with being depressed most of the day, almost each day for a period of two weeks.
